# Quantification of the Effect of Pressure Wire Drift on the Diagnostic Performance of Fractional Flow Reserve, Instantaneous Wave-Free Ratio, and Whole-Cycle Pd/Pa

**DOI:** 10.1161/CIRCINTERVENTIONS.115.002988

**Published:** 2016-04-19

**Authors:** Christopher M. Cook, Yousif Ahmad, Matthew J. Shun-Shin, Sukhjinder Nijjer, Ricardo Petraco, Rasha Al-Lamee, Jamil Mayet, Darrel P. Francis, Sayan Sen, Justin E. Davies

**Affiliations:** From the International Centre for Circulatory Health, National Heart & Lung Institute, Imperial College NHS Trust, United Kingdom.

**Keywords:** diagnosis, diastole, fractional flow reserve, myocardial, physiology, rest

## Abstract

**Background—:**

Small drifts in intracoronary pressure measurements (±2 mm Hg) can affect stenosis categorization using pressure indices. This has not previously been assessed for fractional flow reserve (FFR), instantaneous wave-free ratio (iFR), and whole-cycle distal pressure/proximal pressure (Pd/Pa) indices.

**Methods and Results—Four hundred forty-seven:**

stenoses were assessed with FFR, iFR, and whole-cycle Pd/Pa. Cut point values for significance were predefined as ≤0.8, <0.90, and <0.93, respectively. Pressure wire drift was simulated by offsetting the distal coronary pressure trace by ±2 mm Hg. FFR, iFR, and whole-cycle Pd/Pa indices were recalculated and stenosis misclassification quantified. Median (±median absolute deviation) values for FFR, iFR, and whole-cycle Pd/Pa were 0.81 (±0.11), 0.90 (±0.07), and 0.93 (±0.06), respectively. For the cut point of FFR, iFR, and whole-cycle Pd/Pa, 34.6% (155), 50.1% (224), and 62.2% (278) of values, respectively, lay within ±0.05 U. With ±2 mm Hg pressure wire drift, 21% (94), 25% (110), and 33% (148) of the study population were misclassified with FFR, iFR, and whole-cycle Pd/Pa, respectively. Both FFR and iFR had significantly lower misclassification than whole-cycle Pd/Pa (*P*<0.001). There was no statistically significant difference between the diagnostic performance of FFR and iFR (*P*=0.125).

**Conclusions—:**

In a substantial proportion of cases, small amounts of pressure wire drift are enough to cause stenoses to change classification. Whole-cycle Pd/Pa is more vulnerable to such reclassification than FFR and iFR.

**WHAT IS KNOWN**

- Pressure wire drift decreases the accuracy of transstenotic pressure measurements, potentially leading to stenosis misclassification.
- Pressure wire drift is a common finding at the end of a physiological assessment; ±2 mm Hg of drift is arbitrarily considered the most stringent clinically acceptable threshold in which repeat normalization and physiological assessment are not necessary.
- In clinical practice, indices less vulnerable to stenosis misclassification as a result of drift may be more accurate, quicker, and safer because there is less need to recross lesions for repeat measurements.

**WHAT THE STUDY ADDS**

- The risk of stenosis misclassification with ±2 mm Hg of drift is common in clinical populations and is greatest for values near the cut point.
- Compared with whole-cycle Pd/Pa, FFR, and iFR have similar and superior resilience to pressure wire drift-induced stenosis misclassification change.

Physiological assessment of the hemodynamic impact of a stenosis is recommended by international guidelines.^1,2^ Three pressure-only indices are widely available: fractional flow reserve (FFR), resting whole-cycle distal pressure/proximal pressure (Pd/Pa), and the instantaneous wave-free ratio (iFR). Both FFR and resting whole-cycle Pd/Pa are calculated by averaging mean pressures >3 to 5 beats over the whole cardiac cycle.^3,4^ iFR differs because the analysis is restricted to only the wave-free period of diastole, rather than the whole cardiac cycle,^5–8^ on a beat by beat basis. FFR is measured under conditions of stable pharmacological hyperaemia,^4,9,10^ whereas iFR and Pd/Pa are measured under resting conditions.

**See Editorial by van Lavieren and Piek**

All 3 commonly used indices use pressure sensor-tipped intracoronary wires to quantify the transstenotic pressure gradient. Modern pressure wires offer a high fidelity signal with a digital acquisition. Nevertheless, drift can occur and is often noticed when the pressure wire is withdrawn into the catheter at the end of a physiological assessment.^11^

Drift can be considered a source of error that decreases the accuracy of transstenotic pressure measurements during physiological stenosis assessment. There are multiple causes of drift (Table 1). The Pa measurement is generally through a fluid-filled catheter that is vulnerable to hydrostatic influences. Drift in Pa measurement can be mitigated by adherence to a standardized protocol of best clinical practice during physiological measurement. In contrast, drift in Pd measurement, which can be identified by divergence from the normalized signal, arises from technical characteristics of the Piezoresistive sensor in the pressure wire, which is electric in origin and cannot be fully mitigated by adherence to a standardized protocol. Because even careful practitioners cannot eliminate pressure wire drift, it is important to be aware of the magnitude of its effect on FFR, iFR, and whole-cycle Pd/Pa measurements.

**Table 1.**
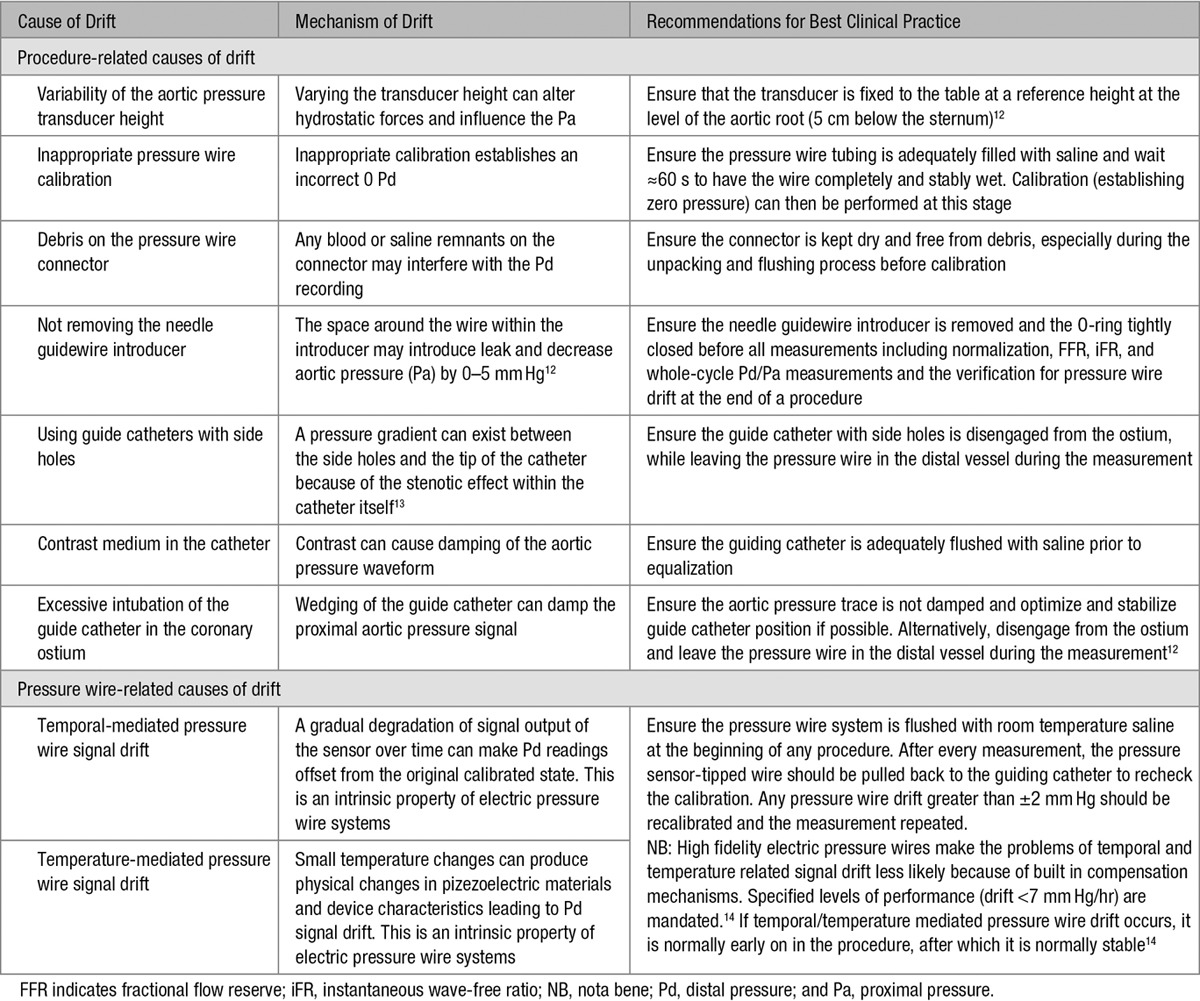
The Causes of Drift During Physiological Stenosis Assessment

When any form of drift is observed at the end of a physiological assessment, repeating the measurement is generally recommended. However, small drifts are difficult to detect and might be assumed to be clinically unimportant. Consequently, even in core laboratory analyses, measurement drift within the range of ±2 mm Hg is considered a clinically acceptable threshold in which repeat normalization and physiological assessment are not necessary.^15,16^

In real world practice, it is unlikely that practitioners apply a more stringent limit on tolerable drift than the ±2 mm Hg used by core laboratories. The extent to which FFR, iFR, and whole-cycle Pd/Pa indices cross a diagnostic threshold with this level of drift has not been formally studied. In this study, we perform the first quantification of the effects of clinically tolerated levels of pressure wire drift on the rates of reclassification with FFR, iFR, and whole-cycle Pd/Pa.

## Methods

### Study Population

This study included 447 patients (447 stenoses; age 62.7±10.1 years; 79% male) scheduled for invasive coronary catheterisation as part of their routine clinical care at Imperial College Healthcare NHS trust, London, United Kingdom. Exclusion criteria were limited to significant valvular pathology, contraindication to adenosine administration (eg, asthma, chronic obstructive pulmonary disease, heart rate<50 beats/min, and systolic blood pressure<90 mm Hg), increased troponin, and weight>200 kg. All subjects gave written informed consent in accordance with the protocol approved by the local ethics committee.

### Procedure and Data Acquisition

The aortic pressure transducer was fixed to the table at a reference height 5 cm below the sternum. Coronary hemodynamic data were obtained using a 0.014-inch electric (Piezoresistive) pressure sensor–tipped wire (Radi PressureWire, St Jude Medical, Minneapolis, MN or PrimeWire Plus, Volcano Corporation, San Diego, CA). The pressure wire was fully flushed with room temperature saline, allowed to wait for 30 to 60 seconds, connected to the pressure wire analyzer interface (ensuring no blood or saline debris on the connector) and zeroed and calibrated according to manufacturer’s instructions. Intravenous heparin was given according to patient weight (70–100 IU/kg) at the start of the procedure and 300 μg of intracoronary nitrates were routinely given before hemodynamic measurements to stabilize epicardial resistance. The pressure wire was then passed into the target vessel via a guiding catheter (without side holes) during diagnostic angiography. Verification of equal signals was made with the sensor located just inside the guiding catheter or in the proximal part of the coronary artery and pressure equalization performed before its advancement distal to the stenosis. Adenosine doses of 140 μg/kg/min (via the femoral vein) or 120 μg (intracoronary) were used to induce vasodilation. Pressure measurements were made at baseline and under maximal pharmacological vasodilation. At the end of the recording, the pressure sensor was returned to the catheter tip to recheck the calibration to ensure that no pressure wire drift had developed. The protocol specified that if pressure wire drift of more than ±2 mm Hg was found, the entire recording was discarded and the process repeated. Thus, for each patient, there was a single valid recording stored for analysis, ie, a single iFR, a single whole-cycle Pd/Pa, and a single FFR.

### Analysis of Hemodynamic Data

Data were analyzed using a custom software package designed with Matlab (Mathworks, Inc., Natick, MA) to calculate physiological stenosis severity by FFR, iFR, and resting whole-cycle Pd/Pa indices. All analyses were performed in a fully automated manner, eliminating the need for manual selection of data time points. Cut point values to define a positive result for FFR, iFR, and resting whole-cycle Pd/Pa were ≤0.8, <0.90, and <0.93, respectively.

### Assessing Measurement Drift

**Figure 1.**
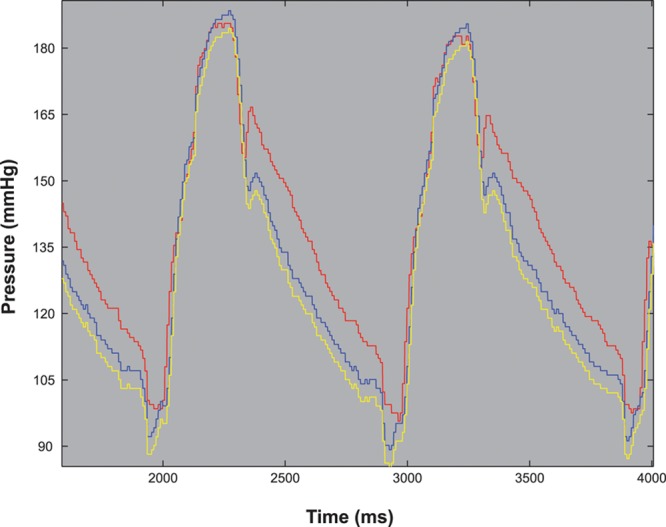
Assessing pressure wire drift. An example of a raw hemodynamic pressure trace with −2 mm Hg of simulated pressure wire drift. Red indicates aortic pressure (Pa), blue indicates distal coronary pressure (Pd), and yellow indicates distal coronary pressure with −2 mm Hg drift.

Pressure wire drift was assessed by offsetting the distal intracoronary pressure trace relative to its original position by 1 mm Hg increments from −2 to +2 mm Hg in the custom software analysis package (Figure 1). Aortic pressure drift was assessed by offsetting the aortic pressure trace by 1 mm Hg increments from −2 mm Hg to +2 mm Hg in the custom software analysis package.

Pressure wire drift of ±2 mm Hg was chosen as this represents a commonly encountered and clinically acceptable range in which repeat normalization and physiological assessment are considered not necessary.^15,16^ FFR, iFR, and resting whole-cycle Pd/Pa values were then recalculated according to the degree of drift and compared with their respective cut point values.

### Assessing Stenosis Misclassification

Stenosis misclassification occurred if recalculated values crossed the threshold for the index (FFR≤0.8; iFR<0.90; whole-cycle Pd/Pa<0.93). The proportion of stenosis misclassification for FFR, iFR, and resting whole-cycle Pd/Pa was plotted against the degree of pressure wire drift across the range of ±2 mm Hg. The cumulative proportion of the cohort that underwent stenosis misclassification was used to compare the resilience of the indices to pressure wire drift.

### Statistical Analysis

The proportion of stenoses that were misclassified as a result of drift between the 3 indices was compared using the McNemar test.^17^ The median absolute deviation was used to measure the spread of the 3 indices, and differences of the spread of values between the groups were compared using the Flinger–Killeen test.^18^ The normality of the distribution of severity measurements was tested using the Shapiro–Wilk test^19^ and skew of the data was assessed with the D’Agostino test.^20^ Statistical analysis was performed using the statistical environment R with the ggplot2 and moments packages.^21^ For all tests, *P*<0.05 was considered significant.

## Results

### Study Population

Patient demographics and lesion characteristics are summarized in Table 2. Overall, mean age was 62.7±10.1 years, and 79% were male. Median (±corrected median absolute deviation) FFR, iFR, and resting whole-cycle Pd/Pa values were 0.81 (±0.104), 0.90 (±0.074), and 0.93 (±0.059), respectively. Overall, 48% (214), 47% (210), and 45% (201) of stenoses were below the cut point value to define a positive result for FFR, iFR, and resting whole-cycle Pd/Pa, respectively. The study population represented a true intermediate cohort, with median values being the same as (or closely related to) the clinical cut point values.

**Table 2.**
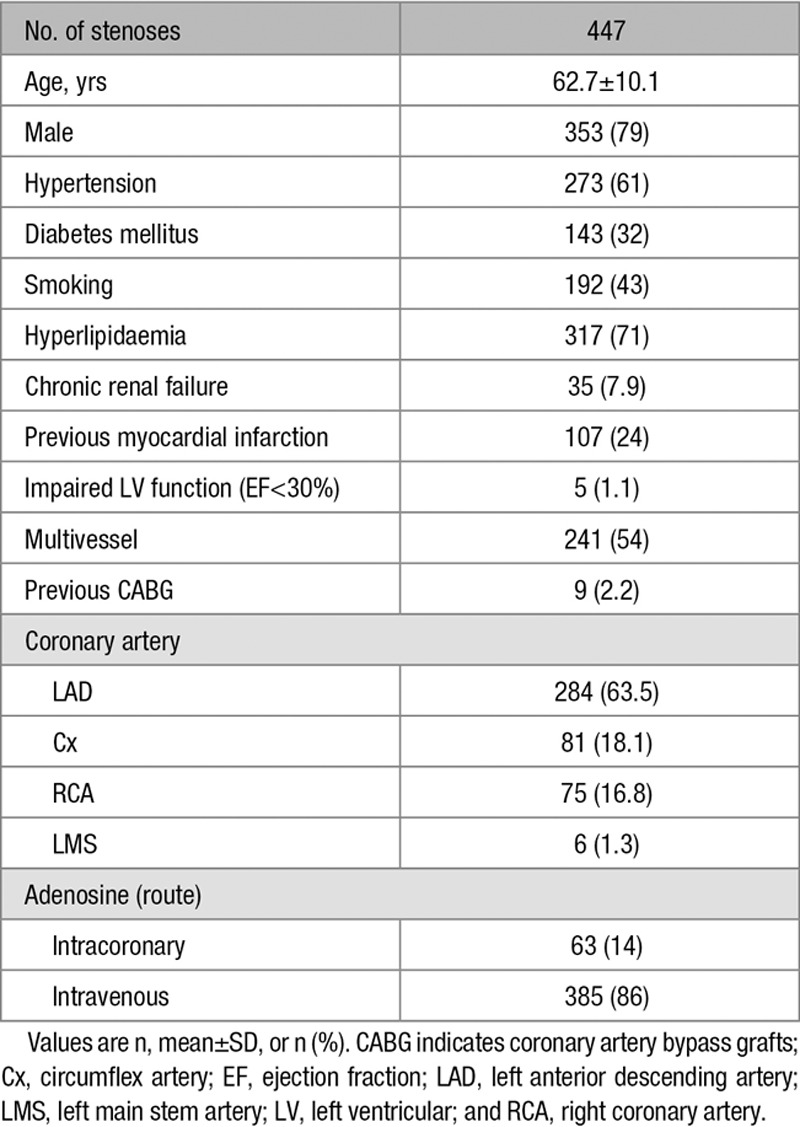
Patient Demographic Data

### Effect of Pressure Wire Drift on Stenosis Misclassification With FFR, iFR, and Whole-Cycle Pd/Pa

**Figure 2.**
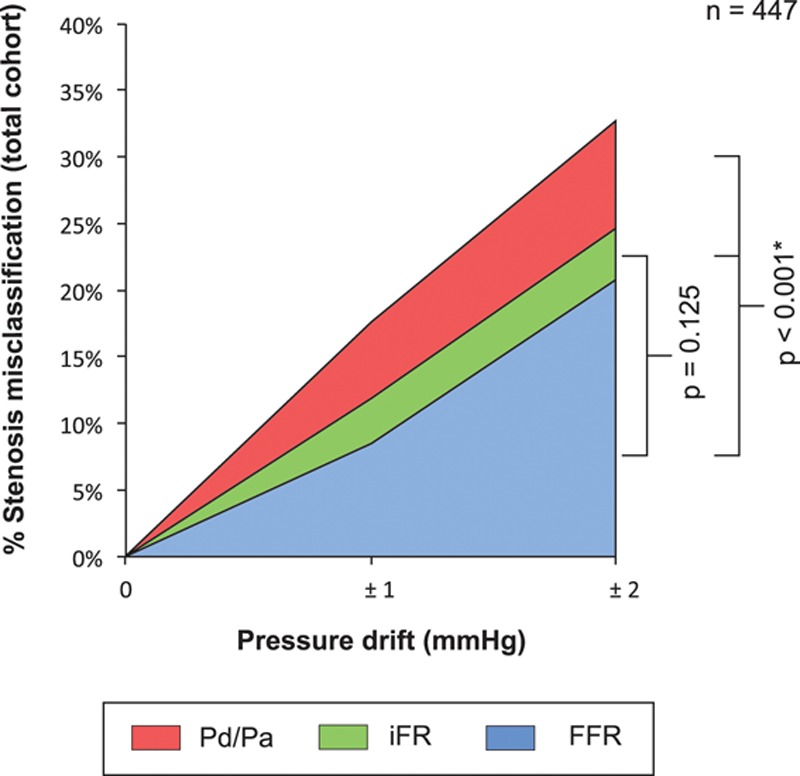
Quantification of the effect of clinically tolerated degrees of pressure wire drift on the diagnostic performance of fractional flow reserve (FFR), instantaneous wave-free ratio (iFR), and whole-cycle distal pressure/proximal pressure (Pd/Pa) in 447 stenoses; 21% (94), 25% (110), and 33% (148) of the total study population were inappropriately misclassified with FFR, iFR, and whole-cycle Pd/Pa, respectively. FFR and iFR were significantly more resilient to stenosis misclassification than whole-cycle Pd/Pa analysis. There was no statistically significant difference in the diagnostic performance of FFR and iFR.

Stenosis misclassification occurred with ±2 mm Hg pressure wire drift across all 3 indices and was commonest with whole-cycle Pd/Pa. Both FFR and iFR had significantly lower proportions of misclassification than whole-cycle Pd/Pa (*P*<0.001). There was no statistically significant difference between the diagnostic performance of FFR and iFR indices (*P*=0.125). Overall, 21% (94), 25% (110), and 33% (148) of the total study population were reclassified with FFR, iFR, and whole-cycle Pd/Pa, respectively (Figure 2). The effect of aortic pressure drift was similar. Overall, 15% (68), 23% (101), and 31% (138) of the total study population were inappropriately misclassified with FFR, iFR, and whole-cycle Pd/Pa, respectively.

### Distribution of Values With FFR, iFR, and Whole-Cycle Pd/Pa

**Figure 3.**
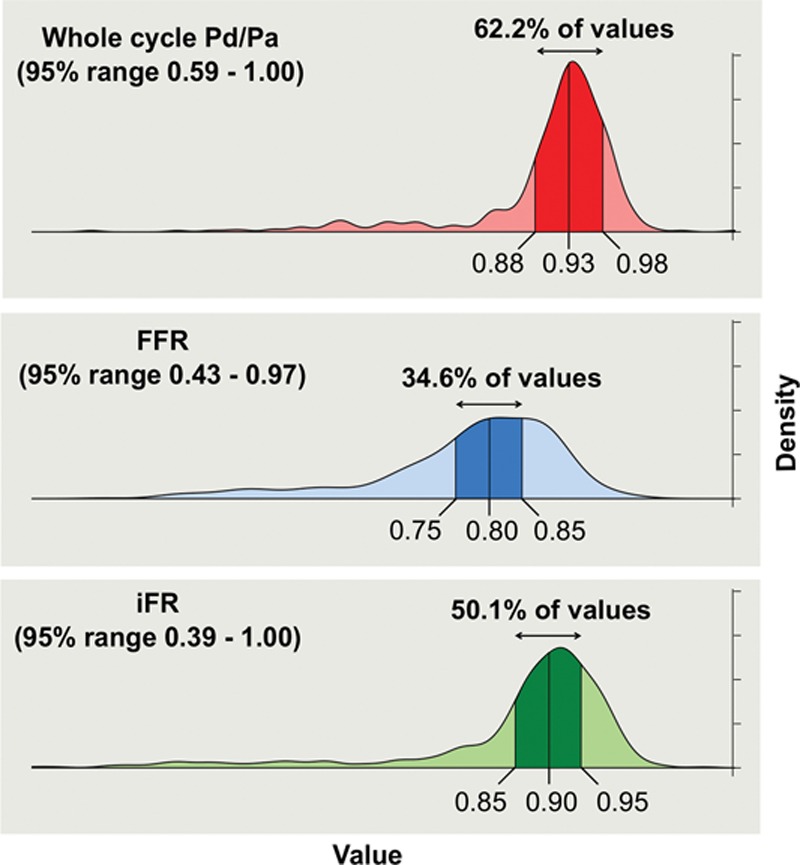
The distribution of values for fractional flow reserve (FFR), instantaneous wave-free ratio (iFR), and whole-cycle distal pressure/proximal pressure (Pd/Pa): density plots show the differing distribution of values with FFR, iFR, and whole-cycle Pd/Pa values for the same 447 stenoses. Solid black lines indicate the cut point value. The highlighted regions indicate values within ±0.05 U of the cut point; 34.6% (155), 50.1% (224), and 62.2% (278) of values lay within ±0.05 U of the cut point for FFR, iFR, and whole-cycle Pd/Pa, respectively.

The distribution of FFR, iFR, and resting whole-cycle Pd/Pa values were all significantly deviated from normality (*P*<0.001 for all 3 indices), with significant negative skew (*P*<0.001 for all three indices). For the same population of stenoses, the dynamic range of values for each pressure-only index was different. This can be expressed as a range of values or by the number of 0.01 U spanned. iFR had the largest dynamic range of values (0.39–1.0, a 62 U spread), compared with FFR (95% range: 0.43–0.97, a 55 U spread) and resting whole-cycle Pd/Pa (95% range: 0.59–1.0, a 41 U spread; *P*<0.001 for all comparisons; Figure 3).

### Proportion of Values Occurring Close to the Cut Point

The risk of stenosis misclassification as a result of clinically accepted degrees of pressure wire drift was greatest for values close to the cut point. 34.6% (155), 50.1% (224), and 62.2% (278) of values lay within ±0.05 U of the cut point for FFR, iFR, and whole-cycle Pd/Pa, respectively.

## Discussion

This is the first study to quantify the effect of commonly accepted pressure wire drift on stenosis classification.

Up to one third of lesions are reclassified with modest pressure wire drift of ±2 mm Hg. Whole-cycle Pd/Pa was more susceptible to reclassification with drift than both FFR and iFR.

### Clinically Accepted Degrees of Pressure Wire Drift Result in Stenosis Misclassification in Up to One Third of Cases

The exact incidence of pressure wire drift is not known. Some insight might be gained from the Multicenter Core Laboratory Comparison of the Instantaneous Wave-Free Ratio and Resting Pd/Pa With Fractional Flow Reserve: the RESOLVE study where 11% of all excluded measurements were because of drift or incorrect calibration.^15^ In clinical practice where physicians may be less aware or have less dedicated time to conduct remeasurments, it is possible that the influence of drift on clinical decision making may be even larger. Physicians recognize that drift is common and so do not report it as an unexpected problem to regulators. This may explain why in the Food and Drug Administration Manufacturer and User Facility Device Experience (FDA MAUDE) database, there are currently only 23 cases of pressure wire drift reported over a 5-year period. Because this seems to be much lower than the incidence of clinically modest drift of ±2 mm Hg, it is not known why those cases and not others were reported. As the denominators are not known, it is not possible to validly calculate or compare rates between different models and manufacturers of wires.

Pressure wire drift can vary during a procedure and may impact some measurements and not others. Therefore, when pressure wire drift is observed after the wire is withdrawn to the ostium of the vessel, there is no reliable way to correct for it, and so it is recommended to repeat the measurements. Indices less vulnerable to drift may therefore be quicker and safer in clinical practice because there is less need to recross lesions for repeat measurements.

There are a variety of proposed thresholds for an acceptable degree of drift that does not require repeat normalization and recrossing of the lesion to remeasure. The industry standard for pressure wire manufacture is less than ±7 mm Hg/hr.^14^ Expert consensus accepts a threshold of less than ±5 mm Hg,^11^ and core laboratory analysis accepts a threshold of ±2 mm Hg.^16^ The experimental methods of determination of these thresholds were not specified, and therefore, it is not know why they are so different.

In our study, we have examined the effect of a level of drift considered acceptable even under the most stringent of these thresholds, namely ±2 mm Hg. Accepting some small level of drift helps the procedure reduce recrossing of lesions. Until now, the effect of this on the diagnostic performance of pressure based physiological indices was not known. Our study indicates that with pressure wire drift of ±2 mm Hg stenosis misclassification can occur in up to one third of patients, in the case of whole-cycle Pd/Pa. The proportion is large because in real world patient groups many values are in the central region near the cut point (Figure 3). In this region, which contains many patients, only a small pressure wire drift is needed to cause misclassification.

### FFR and iFR Are More Resilient Than Whole-Cycle Pd/Pa to Stenosis Misclassification by Drift

**Figure 4.**
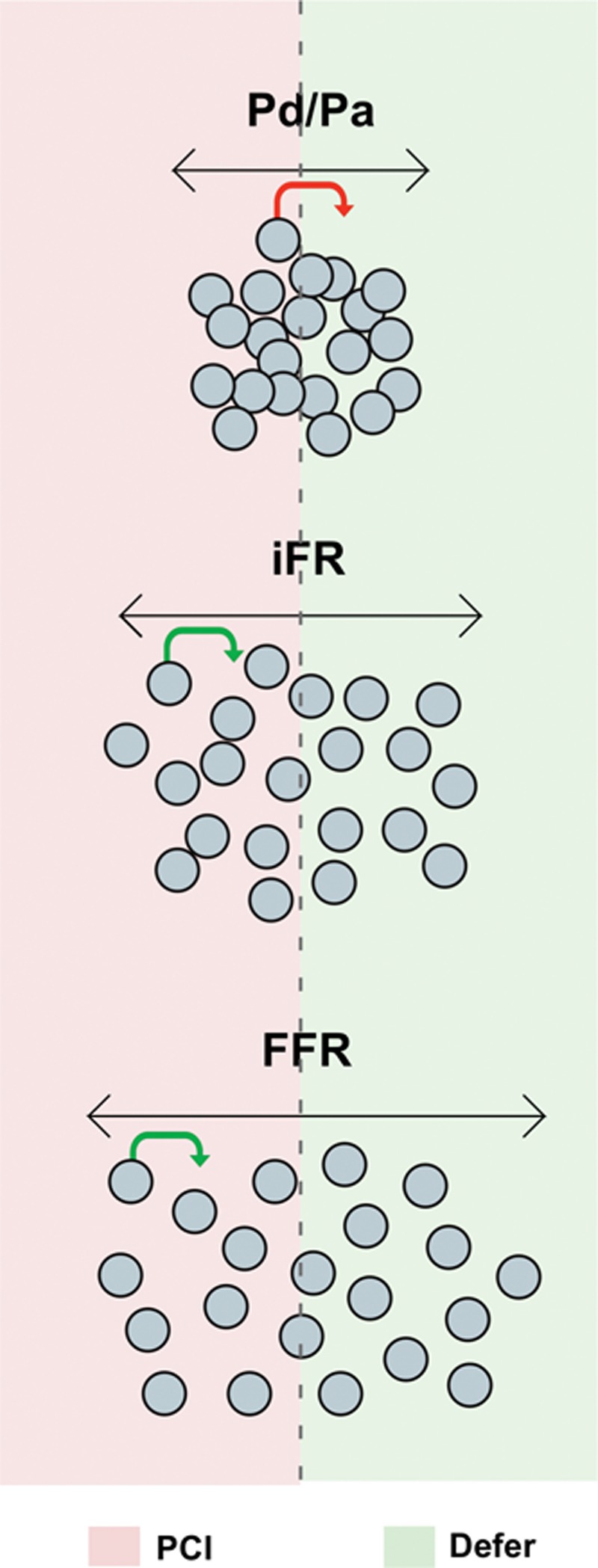
The risk of drift-induced stenosis misclassification is greatest for values near the cut point: A value significantly removed from the cut point will be unlikely to be affected by drift (green arrow), whereas those close to the cut point will be more susceptible to stenosis misclassification (red arrow). FFR indicates fractional flow reserve; iFR, instantaneous wave-free ratio; PCI, percutaneous coronary intervention; and Pd/Pa, distal pressure/proximal pressure.

The same absolute pressure wire drift in mm Hg has the same impact on all 3 indices. Their differences in drift-induced misclassification therefore arise from the different proportion of values that lie close to the cut point (Figure 3). The further an index’s value to its cut point, the less susceptible is the lesion to misclassification from pressure wire drift. In practice, this means indices with fewer values close to the cut point will be more robust when clinically accepted levels of drift occur (Figure 4).

Indices such as FFR and iFR offer more gradations of value to describe a stenosis, using an identical wire. For example, it can become possible to resolve small differences in the index during a pullback along a length of diseased artery,^22,23^ helping identify the hemodynamically most significant parts of the disease. This improvement in signal-to-noise ratio can therefore help target percutaneous coronary intervention to the physiologically most appropriate stenoses.

Our data indicate a similar rate of misclassification by pressure wire drift for FFR and iFR. This is in line with other studies indicating that pharmacological hyperaemia (FFR) and the inherent increase in coronary flow in the wave-free period of diastole (iFR), both perform equally well when compared with other quantitative tests of ischemia.^6,24–26^

By selectively measuring during the wave-free period, iFR uses an intrinsic phase of the cardiac cycle where myocardial resistance is naturally most stable and lowest^5^ without the need for pharmacological hyperaemia. This leads to assessment over a period where coronary flow velocity is highest and the pressure gradient greatest in the resting state.^5,6^ Greater flow accentuates transstenotic pressure gradients and ratios, thereby providing greater discrimination between stenosis severities,^8^ a wide dynamic range of values,^27^ and permits resilience to pressure wire drift–induced stenosis misclassification that is comparable with FFR.

The recognition that resting physiology could provide an alternative to hyperaemic measures^28^ continues to gain significant interest. Both conceptually and practically, this approach is attractive as it removes the need for hyperaemia, may promote physiology usage, reduce procedural costs and time, and eliminate the side effects associated with adenosine.^5^

### Limitations of Previous Comparisons Between FFR, iFR, and Whole-Cycle Pd/Pa

Although recognized as a commonly encountered problem, the effect of pressure wire drift on stenosis misclassification has not been considered in any core laboratory analyses to date. Until now, comparisons of diagnostic performance have been largely based on the linear correlation between values measured with 1 index and another.^15,16^ For application to clinical practice, this could be extended by a consideration of the relative vulnerabilities of the difference in indices to misclassification from pressure wire drift.

### Limitations

This study did not include measurement of real drift but rather simulated drift form the real world raw pressure tracings (Figure 1). As real drift is not reproducible, it would not be possible to perform a comparative systematic analysis. By simulating drift using standard engineering approaches, with real world raw pressure tracings, this allowed a physiologically representative data set to be assessed.

When assessing pressure drift based on an absolute mm Hg value, patients presenting with low aortic pressures will be influenced proportionally more compared with patients with high aortic pressures. This is mathematical phenomenon and therefore common to each index.

FFR is recognized to be critically dependent on the response to hyperaemia. Despite all patients receiving high doses of adenosine, it is feasible that the dynamic range could have been even larger if additional hyperaemic agents had been used.^29,30^ However, these levels of hyperaemic agents are similar to or exceed the levels administered in large clinical outcome studies and therefore are representative of a guideline defining population.

FFR alone is currently the only technique with significant evidence to attain recognition in the clinical guidelines.^1,2^ In contrast, iFR and resting whole-cycle Pd/Pa have no clinical outcome data to support their use. This means that until the results of large clinical outcome trials comparing FFR and iFR (such as Functional Lesion Assessment of Intermediate Stenosis to Guide Revascularisation [DEFINE-FLAIR, NCT02053038] and Evaluation of iFR vs FFR in Stable Angina or Acute Coronary Syndrome [iFR Swedeheart, NCT02166736]) are reported, our results should be considered physiologically descriptive but not disruptive of the established FFR technique.

This study was limited to the analysis of the effects of pressure wire drift on FFR, iFR, and whole-cycle Pd/Pa measured with electric (piezoresistive) sensor-tipped pressure wires. Optical sensors use a different method for quantifying intracoronary pressure gradients, with pressure changes measured by using light to assess the width of a cavity within the sensor. Because piezoresistive sensors are susceptible to thermal and temporal signal drift,^31^ this provides optical sensors a theoretical advantage. However, the larger size of optical sensors may limit their application to all forms of assessment and further studies are required to evaluate fully the clinical value of optical sensor technology.^31^

### Conclusions

Clinically accepted pressure wire drift (±2 mm Hg) can cause stenosis misclassification with all frequently used pressure-only indices of stenosis severity. The risk of misclassification is common in clinical populations and is greatest for values near the cut point. Whole-cycle Pd/Pa is the most susceptible index because the majority of values lie close to the cut point. FFR and iFR are comparably less susceptible because fewer values lie close to the cut point. This study would support clinical practice to be as stringent as the strictest core laboratory protocol, which is to not accept data where the pressure wire drift exceeds 2 mm Hg.

## Sources of Funding

This research was supported by the National Institute of Health Research (NIHR) Biomedical Research Centre based at Imperial College Healthcare National Health Service (NHS) Trust and Imperial College London. Dr Cook (MR/M018369/1), Dr Sen (G1000357), and Dr Nijjer (G110043) are Medical Research Council Fellows. Dr Davies (FS/05/006), Dr Francis (FS 10/038), Dr Petraco (FS/11/46/28861), and Dr Shun-Shin (FS/14/27/30752) are British Heart Foundation Fellows.

## Disclosures

Dr Davies is a consultant for Volcano Corporation and coinventor of iFR. Drs Davies and Mayet have intellectual property interests in iFR Technology. Drs Cook, Petraco, Sen, and Nijjer have received travel support from Volcano Corporation. The other authors report no conflicts.
